# Impact of the COVID-19 pandemic on corticosteroid injection services: A National Survey of Members of the British Society of Skeletal Radiologists (BSSR)

**DOI:** 10.1259/bjr.20210327

**Published:** 2022-07-08

**Authors:** Danoob Dalili, Rory Fairhead, Asimenia Mermekli, Joseph Papanikitas, James Teh, Richard Hughes, Daniel Fascia, David McKean

**Affiliations:** 1 South West London Elective Orthopaedic Centre (SWLEOC), Dorking Road, Epsom, London, United Kingdom; 2 Epsom and St Helier University Hospitals NHS Trust, Dorking Road, Epsom, London, United Kingdom; 3 Pembroke College, Oxford University Medical School, Oxford, UK; 4 Radiology Department, Stoke Mandeville Hospital, Buckinghamshire Healthcare NHS Trust, Aylesbury, United Kingdom; 5 Radiology Department, Nuffield Orthopaedic Centre, Oxford University Hospitals NHS Trust, Oxford, United Kingdom; 6 Radiology Department, Harrogate and District NHS Foundation Trust, Harrogate, United Kingdom

## Abstract

**Objective:**

To describe the restructuring of services by British radiologists in response to evolving national guidelines and highlight the impact of the COVID-19 pandemic on the treatment of musculoskeletal (MSK) conditions.

**Methods:**

An online anonymised survey was distributed via the British Society of Skeletal Radiology (BSSR) members forum in November 2020. Responses were collated using a standardised Google form including 21 questions.

**Results:**

135 members of the BSSR completed the survey. 85% of respondents stopped performing corticosteroid injections (CSI) during the initial lockdown of the pandemic. This was primarily influenced by national guidelines. The majority of respondents initially abstained from offered CSI procedures, then by November 2020, 69% of respondents were providing CSI for high and low risk patients, 23% were only providing CSI for low-risk patients with 8% still not performing any CSI. 40% of respondents reported routinely obtaining specific written consent regarding the risk of COVID-19. Approximately, 11,000 CSI were performed by respondents between March and November 2020 with no reported significant COVID-19-related complications. Over 80% of BSSR members reported that the number of CSI procedures that they performed dropped by more than 80% compared to usual. 73% of respondents reported an increased backlog of patients awaiting treatment. The average waiting time for routine outpatient CSI treatment was > 12 weeks in 53% of responses, compared to 34% the previous year.

**Conclusion:**

The COVID-19 pandemic has had a significant impact on the clinical practices of MSK radiologists in the UK. Our survey highlights the rapid response of BSSR members as national guidelines evolved. Currently, the majority of respondents are performing CSI for musculoskeletal conditions when clinically indicated, with enhanced consent. However, the pandemic has resulted in increased waiting times – delaying the treatment of patients who may be suffering with significant pain and disability. Further research is warranted to provide guidance around both service recovery and provision of CSI around COVID-19 vaccination schedules.

**Advances in knowledge:**

BSSR members responded rapidly to changing guidelines during the COVID-19 pandemic. The majority of respondents are currently performing CSI when clinically indicated. The pandemic has resulted in a significant increase in waiting times which will have a significant impact on UK musculoskeletal services.

## Introduction

Musculoskeletal image-guided interventional procedures represent a core service offered by musculoskeletal radiologists globally.^1^ Corticosteroid injections (CSI) play an instrumental role in these services, offering treatment for a wide range of degenerative and inflammatory conditions.^2^

At the onset of the COVID-19 pandemic, UK national guidelines were published cautioning against the use of CSI, in part due to the well-recognised immunosuppressive effects of CSI and in part to reduce patient footfall during the first peak of UK infections.^3–10^ The British Society of Skeletal Radiologists (BSSR) was one of the first societies to publish guidelines on corticosteroid injection administration, issuing its guidelines in March 2020.^3^ In March 2020, the British Society for Rheumatology advocated “extreme caution and serious discussion with the patient prior to proceeding [sic] CSI, as well as use of minimum doses to achieve pain relief”.^5^ This was echoed by an expert panel of pain physicians, psychologists and researchers from North America and Europe^11^ who concluded that intra-articular steroids administered during the COVID-19 pandemic may increase potential for adrenal insufficiency and altered immune response, hypothetically increasing the risk of contracting the virus. It was also suggested that changing the dose and type of steroid should be considered. In addition, the Faculty of Pain Medicine of the Royal college of Anaesthetists issued guidance in March. This recommended that CSI must not be undertaken in individuals with active infections and that use of steroids should be exercised with caution, clarifying the risk – benefit balance on an individual, case-by-case basis. In addition, patients should be fully aware of the potential increased risk, the lack of clear evidence, and be engaged in decision-making. Likewise, individual units should consider the risk and benefits of such injections and under which circumstances they will continue using them during the current clinical conditions.^9^

Certain cohorts have been identified as being high risk for contracting and subsequently reacting to the virus, with relatively prolonged recovery and higher mortality rates. These include immunocompromised patients,^1,5,7,11–13^ patients over the age of 70, those with diabetes mellitus, ischaemic heart disease, chronic respiratory disease and/or hypertension, high body mass index and patients of black and minority ethnic groups as well as those presenting with low vitamin D levels.^10,11,13,14^ Such COVID-19 vulnerable groups constitute a large percentage of patients presenting with chronic musculoskeletal conditions which respond to CSIs and therefore present to musculoskeletal clinics.

Since the onset of the pandemic, the variable effect of acute and chronic corticosteroids on COVID-19 outcomes has been further investigated. Much of the pathology of severe acute COVID-19 is driven by the consequences of unconstrained host inflammatory response.^15^ Corticosteroids have been shown to be an effective treatment for COVID-19 in the acute setting, with 6 mg of dexamethasone (equivalent to 40 mg daily of prednisone) daily for up to 10 days reducing mortality from 25.7 to 22.9% (rate ratio [RR] 0.83, 95% CI 0.75–0.93).^16^ It was also been noted that there is a significant under-representation of patients with asthma and chronic obstructive pulmonary disease (COPD) in patients hospitalised with COVID-19—hypothesised to be related to the intermittent use of inhaled glucocorticoids.^17^ Finney et al^18^ demonstrated using human and animal *in vitro* and *in vivo* disease models that inhaled corticosteroid (ICS) administration attenuates pulmonary expression of the SARS-CoV2 viral entry receptor—angiotensin-converting enzyme (ACE)−2 which is postulated to reduce susceptibility to COVID-19 infection. In addition, recent data have suggested that early administration of inhaled budesonide reduces the likelihood of needing urgent medical care and reduces time to recovery following early COVID-19 infection.^19^ In contrast, registry data suggest that chronic glucocorticoids may increase the odds of hospitalisation for COVID-19 in patients with rheumatic disease, with an adjusted odds ratio (aOR) of 2.05 (95% CI 1.06–3.96) in those taking 10 mg or more prednisone equivalent per day.^20^ While it is clear that further research is required to investigate the dichotomy between the effects of acute and chronic steroids in COVID-19,^15^ the small studies done to date have not shown a higher infection rate or increased risk of adverse outcomes in patients following CSI.^2,14^

The BSSR is the Special Interest Group of the Royal College of Radiologists representing British Musculoskeletal Radiologists. This survey outlines the response of BSSR members to evolving national guidelines 10 months following arrival of the pandemic in the UK and highlights the impact of COVID-19 on the provision of CSI services and the impact of the restructuring of these services on waiting lists for CSI in the UK.

## Methods and materials

In line with the National Institute’s for Health Research (NIHR) guidelines, institutional review board approval was not required for this survey, as the survey did not involve directly any patient-related data.^21^

A questionnaire was developed by five BSSR members in collaboration with two members of the BSSR Executive Committee who had a primary role in developing the national guidelines issued in March 2020.^3^ In line with previous studies, an online Google form^22^ was used to form, disseminate and collect the results of the survey. The anonymous survey was composed of 21 questions; the full list of questions and answers are displayed in Table 1. Questions addressed the variations in clinical practice in both the National Health Service (NHS) and private sector, the influencing factors and impact on workload. The majority of questions were multiple choice with one final question requesting a free text response.

**Table 1.** Survey questions

Question	Answer
1.Do you perform CSI in your routine clinical practice?	Yes: 99% (134/135)
	No: 1% (1/135)
2.Do you perform CSI in NHS practice, private practice or both?	NHS: 30% (40/135)
	Private practice: 4% (5/135),
	Both: 64% (87/135),
	Abstained: 2% (2/135)
3.Did you stop performing CSI during the initial phase of the pandemic? Initial phase of the pandemic approximating February-June 2020. (High risk patients would include patients over the age of 70, those with diabetes mellitus, ischaemic heart disease, chronic respiratory disease and hypertension).	Stopped all CSI: 85% (115/135)
	Only injected low risk patients: 3% (8/135)
	Injected low and high-risk patients: 5% (12/135)
4.Were changes in your CSI practice at the onset of the pandemic influenced by personal opinion, local guidelines or national guidelines? (please tick all that apply)	National guidelines: 29% (39/135)
	Local guidelines: 7% (10/135)
	Personal opinion: 7% (9/135)
	Local guidelines & National guidelines: 23% (31/135)
	National guidelines & Personal opinion: 4% (6/135)
	Local guidelines & Personal opinion: 4% (6/135)
	National guidelines, Local guidelines & Personal opinion: 24% (33/135)
	N/A 1% (1/135)
6.Were you aware of the BSSR guidance on CSI published 19th March 2020?	Yes: 95% (128/135)
	No: 4% (6/135)
	Abstained: 1% (1/135)
5.Were you aware of the British Society for Rheumatology, British Association of Orthopaedics, British Association of Spinal Surgeons, Royal College of General Practitioners, British Society of Interventional Radiology, Faculty of Pain Medicine, British Pain Society and Chartered Society of Physiotherapy guidance published 16 June 2020?	Yes: 85% (115/135)
	No: 14% (19/135)
	Abstained: 1% (1/135)
7.Are you performing CSI in your current clinical practice? (High risk patients would include patients over the age of 70, those with diabetes mellitus, ischaemic heart disease, chronic respiratory disease and hypertension).	Yes- I currently perform CSI for musculoskeletal conditions in low risk patients when clinically indicated: 23% (31/135)
	Yes- I currently perform CSI for musculoskeletal conditions in low and high-risk patients when clinically indicated: 68% (92/135)
	No; 8% (11/135)
	N/A; 1% (1/135)
8.Do you consent patients for the potential risk of CSI during the COVID-19 pandemic?	Yes- verbal consent obtained; 39% (53/135)
	Yes- written consent obtained using COVID specific consent form; 40% (54/135)
	Yes- written consent obtained using generic consent form; 18% (24/135)
	No specific consent for risk of CSI during the pandemic; 2% (2/135)
	N/A; 1% (2/135)
9.Have you reduced the dosage of steroid used for any CSI you perform?	Yes: 31% (42/135)
	No: 68% (92/135)
	Abstained: 1% (1/135)
10.In your current clinical practice, do you require patients to have a negative COVID-19 test (PCR, lateral flow etc) prior to receiving a CSI?	Yes: 10% (13/135)
	No: 90% (121/135)
	Abstained: 1% (1/135)
11.In your current practice, do you advise that patients shield following receiving a CSI?	Yes: 33% (45/135)
	No: 64% (87/135)
	Abstained: 2% (3/135)
12.Since the onset of the COVID-19 pandemic, approximately how many CSI have you performed in all clinical settings?	0; 6% (8/135)
	0–50; 40% 54/135)
	50–100; 25% (34/135)
	100–150; 8% (11/135)
	150–200; 9% (12/135)
	200–300; 6% (8/135)
	300–400; 2% (3/135)
	>400; 3% (4/135)
	N/A; 1% (1/135)
13.Of patients you have treated with CSI, are you aware of any who have suffered an adverse clinical outcome related to COVID-19 infection?	Yes (we have audited the majority of these patients’ clinical outcomes); 1% (1/135)
	No (we have audited a proportion of these patients’ clinical outcomes); 4% (6/135)
	No (we have audited the majority of these patients’ clinical outcomes); 7% (9/135)
	No (we have not formally audited patients’ clinical outcomes); 87% (118/135)
	N/A; 1% (1/135)
14.If you are aware of any patients who have suffered adverse clinical outcomes related to COVID-19 infection following CSI please give further details below (*e.g.* “ICU admission 2 weeks following CSI”).	Yes; 0% (0/135)
	Not aware of any; 100% (135/135)
15.What % of CSI are you currently performing compared to the same time last year?	<20%; 21% (29/135)
	20–40%; 17% (23/135)
	40–60%; 25% (34/135)
	60–80%; 15% (20/135)
	80–100%; 19% (25/135)
	>100%; 1% (2/135)
	N/A; 1% (2/135)
16.Has the pandemic resulted in a change in the backlog of patients awaiting CSI for musculoskeletal conditions in your region?	Increased backlog of patients; 73% (99/135)
	Unchanged backlog of patients; 13% (17/135)
	Reduced backlog of patients; 13% (18/135)
	N/A; 1% (1/135)
17.What was the approximate mean waiting time for a routine outpatient CSI for musculoskeletal conditions in your local NHS department this time last year?	<6 weeks; 32% (43/135)
	6–12 weeks;32% (43/135)
	12–18 weeks; 20% (27/135)
	18–24 weeks; 5% (7/135)
	24–30 weeks; 4% (6/135)
	>30 weeks; 5% (7/135)
	N/A; 1% (2/135)
18.What is the approximate mean waiting time for a routine outpatient CSI for musculoskeletal conditions in your local NHS department currently?	<6 weeks; 23% (31/135)
	6–12 weeks; 23% (31/135)
	12–18 weeks; 15% (20/135)
	18–24 weeks; 12% (16/135)
	24–30 weeks; 9% (12/135)
	>30 weeks; 16% (21/135)
	N/A; 3% (4/135)
19.In what region of the country are you based?	England: South East; 16% (22/135)
	England: South West; 18% (24/135)
	England: North West; 13% (18/135)
	England: North East; 1% (2/135)
	England: London; 19% (25/135)
	England: Yorkshire and the Humber; 7% (10/135)
	England: East Midland; 1% (2/135)
	England: East of England; 4% (6/135)
	England: West Midlands; 8% (11/135)
	Scotland; 8% (11/135)
	Wales; 3% (4/135)
20.If there are any other observations regarding the use of CSI for MSK conditions during the pandemic which you would like to share please add these below:	Free text responses.
**Question**	**Answer**
1.Do you perform CSI in your routine clinical practice?	Yes: 99% (134/135)


BSSR, British Society of Skeletal Radiologists; CSI, corticosteroid injections.

On 20 November 2020, just prior to the commencement of the annual BSSR members meeting (held virtually), invitations were sent via an announcement on the online forum as well as via email distribution to all active full BSSR members. After 1 week, a reminder email was sent to members. The survey was concluded on the 4 December 2020, 14 days after initiation.

### Data statistical analysis

All responses and comments were electronically collected by Google forms in an electronic spreadsheet (Microsoft Excel, Microsoft, Redmond, VA). Data were then tabulated and analysed by two radiologists with experience in survey studies (DM & DD). Descriptive statistics were used, with means and percentages to express the results.

## Results

135 of 458 BSSR members completed the survey, a response rate of 29%, representing most regions within the United Kingdom; England, Scotland, and Wales (Table 1).

Almost all responding BSSR members perform CSI in their routine clinical practice (134/135; 99%), whether solely in the NHS (40/135; 30%), in their private practice (5/135; 4%), or in both (87/135; 64%).

**Figure 1.**
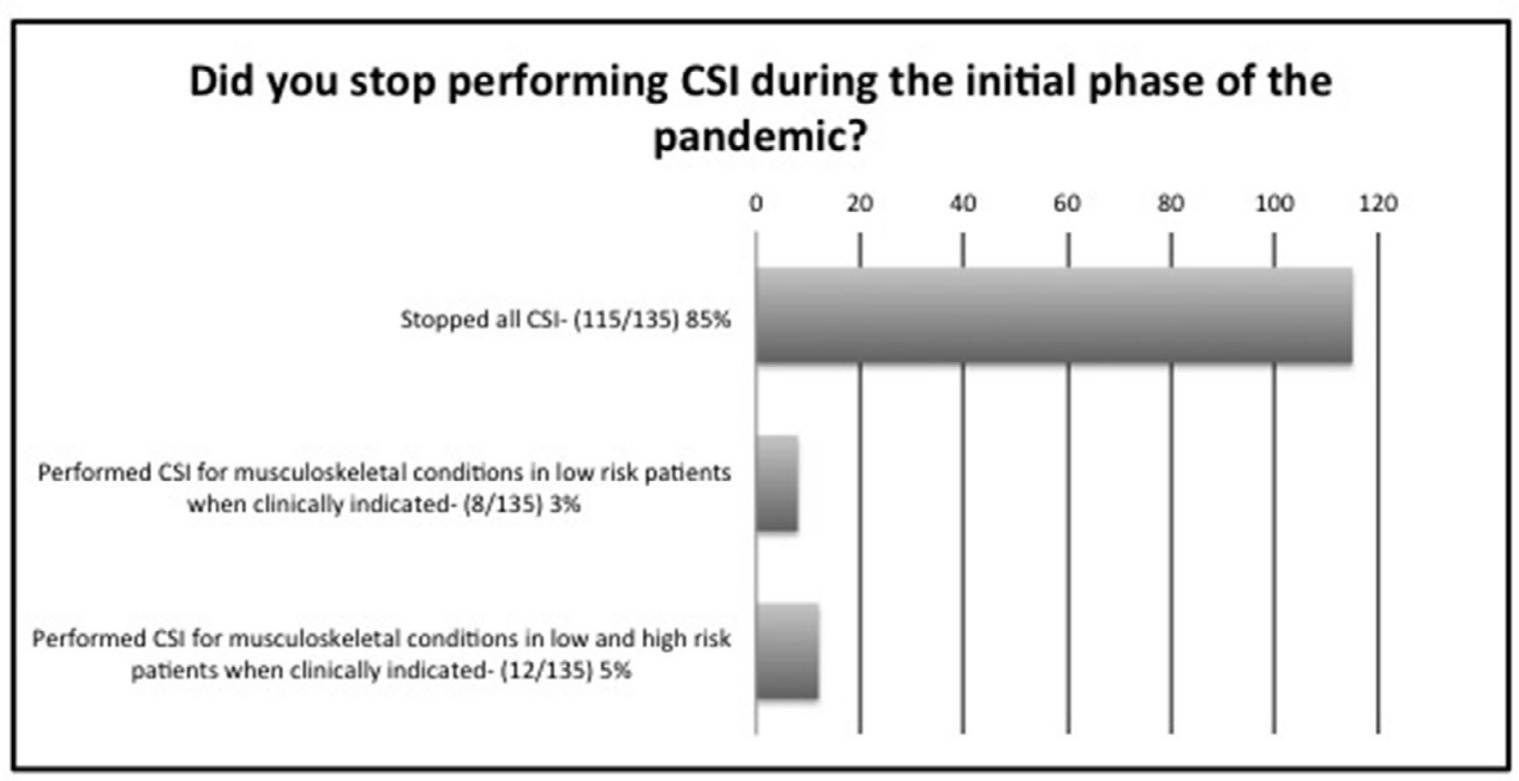
Survey of BSSR members practice between February and June 2020. CSI, corticosteroid injections.

**Figure 2.**
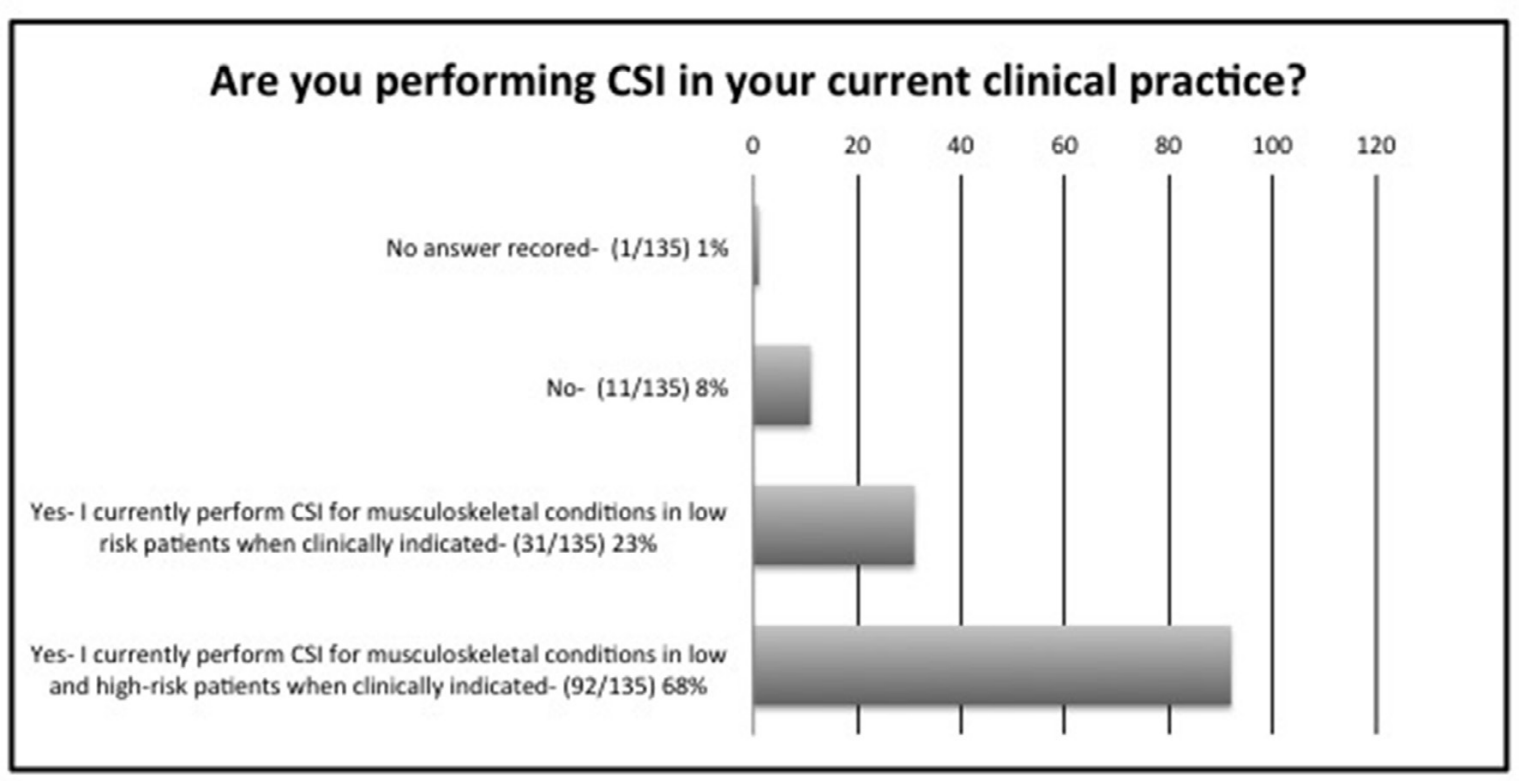
Survey of BSSR members practice in November 2020. CSI, corticosteroid injections

During the initial phase of the pandemic, *i.e.* between February and June 2020, 115/135 (85%) respondents stopped all CSI, 8/135 (6%) only injected low risk patients, whereas 12/135 (14%) injected both low and high risk patients, if clinically indicated by the referring clinician (Figure 1). High risk patients were defined to patients over the age of 70, those with diabetes mellitus, ischaemic heart disease, chronic respiratory disease or hypertension. The patterns changed by November 2020, which coincided with tier 3 and 4 lockdowns throughout the UK, where 31/135 (23%) performed CSI for musculoskeletal conditions in low risk patients, 92/135 (68%) for low and high risk patients, whilst only 11/135 (8%) reported that they did not perform any CSI (Figure 2). 128/135 (95%) were aware of the BSSR guidance on CSI published on 19 March 2020^3^ and 115/135 (85%) were also aware of the British Society for Rheumatology, British Association of Orthopaedics, British Association of Spinal Surgeons, Royal College of General Practitioners, British Society of Interventional Radiology, Faculty of Pain Medicine, British Pain Society and Chartered Society of Physiotherapy guidance published on 16 June 2020.^23^

Factors influencing CSI practice at the onset of the pandemic included: national guidelines (39/135; 29%)**,** local guidelines (10/135; 7%), both local guidelines & national guidelines (31/135; 23%), national guidelines, local guidelines & personal opinion (33/135; 24%), personal opinion (9/135; 7%), national guidelines & personal opinion (6/135, 4%), local guidelines & personal opinion (6/135; 4%) whilst one radiologist felt it was non-applicable (1/135; 1%).

Regarding the consenting process, 53/135 (39%) obtained verbal consent that included the potential risk of CSI during the COVID-19 pandemic, 54/135 (40%) obtained written consent using COVID-19 specific consent form, 24/135 (18%) obtained written consent using a generic form, 2/135 (1%) did not include specific consent for risk of CSI during the pandemic; whilst one radiologist (1%) abstained.

More than two-thirds of survey responders did not change the dosage of steroid used for any CSI procedures 92/135 (68%), whilst 42/135 (31%) reduced the overall dose administered.

Since the onset of the COVID-19 pandemic the average CSI procedures performed in all clinical settings by individual responding radiologists were reported to be as follows; 0 procedures 6% (8/135), 0–50 procedures 40% (54/135), 50–100 procedures 25% (34/135), 100–150 procedures 8% (11/135), 150–200 procedures 9% (12/135), 200–300 procedures 6% (8/135), 300–400 procedures 2% (3/135), >400 procedures 3% (4/135), N/A 1% (1/135). There was a significant change in the volume of CSI procedures performed compared to the same time the year before: 21% (29/135) of respondents reported a drop to <20% compared to pre-pandemic workload; 17% (23/135) reported a drop to 20–40%; 25% (34/135) reported a drop to 40–60%; 15% (20/135) reported a drop to 60–80%; 19% (25/135) reported a drop to 80–100%; whilst two radiologists reported an increase - >100%; 1% (2/135).

COVID-19-related adverse effects included any of COVID-19 symptoms defined by the World Health Organisation (WHO) and NHS England.^24,25^ Major complications included those requiring hospital admissions. When asked about reported complications in patients treated with CSI during the pandemic, one radiologist reported adverse outcomes following auditing the majority of these patients’ clinical outcomes, 7% (9/135) who said they had audited the majority of these patients’ clinical outcomes but there were no adverse outcomes compared to 4% (6/135) who only managed to audit a proportion of patients. 87% (118/135) did not audit their patients’ outcomes but stated that they were not aware of any adverse outcomes amongst their patients. However, the results were changed when asked specifically regarding the details of those outcomes, as a 100% (135/135) of respondents stated that they were not aware of any adverse clinical outcomes related to COVID-19 infection following CSI.. No further details were included in the free text.

Regarding a change in the backlog of patients awaiting CSI for musculoskeletal conditions in their respective regions, 73% (99/135) felt that the pandemic increased their backlog of patients; 13% (17/135) reported an unchanged backlog of patients; whilst 13% (18/135) reported a reduced backlog of patients. A free text section was provided to guide future work and provide a space for accounts of personal experience.

## Discussion

Our survey succeeded in capturing opinions of specialised expert MSK radiologists across the UK, achieving countrywide representation of events taking place during 2020, despite some areas being affected more than others. With regards to the level of guidance most linked to change in practices, national guidelines were a driver for change in practices in 52%, and together with local guidelines both guidelines influenced decision-making in 23%. This was further reinforced by personal opinion in another 24% (based on own experiences or from literature, discussion with colleagues etc).

During the initial phase of the pandemic and until June 2020, 115/135 (85%) of respondents stopped all CSI whilst 8/135 (6%) only injected low risk patients, confirming their adherence to BSSR guidance (Figure 1). This was concordant with national and international guidelines issued by other practitioners, who were also performing CSI over the same time duration^3,4,7,9,10^ and evidenced multidisciplinary success in maintaining standardised care during times of uncertainties. The pattern and volume of CSI services changed by winter, as recorded at the time of the survey, where 23% of respondents performed CSI for musculoskeletal conditions in low risk patients when clinically indicated, whilst 68% performed CSI for musculoskeletal conditions in low and high risk patients when clinically indicated and only 6% abstained from CSI procedures. This may be related to further published evidence, which dissipated concerns regarding any adverse effects linked to CSI.^2,6,10,14^ In our survey, when asked for known details regarding patient outcomes, 100% (135/135) of respondents stated that they were not aware of any adverse clinical outcomes related to COVID-19 infection in any of their patients in whom they offered CSI, although this had not been formally audited in the majority of cases.

Warning patients about the potential increase risk of COVID due to steroids in the consenting process was deemed important by 97% of radiologists, and has been reported to not influence decision to proceed with CSI in the majority of patients.^6^ We advocate that patients should continue to be offered CSI where indicated, whilst emphasising that adequate and clearly documented counselling is fundamental.

Due to the relatively small doses administered during CSI in MSK services, the majority of respondents (68%), did not change their practices in terms of dosage, whilst some (31%) reduced the overall dose administered.

Since the onset of the COVID-19 pandemic the average number of CSI procedures performed in all clinical settings per respondent was reported to be less than 100 procedures in 2020 to the date of our survey in 65% of respondents, which was a drop of more than 60% of workload in 63% of those responding to the survey, compared to 2019. That combined with an on average increase in demand year on year by 10–40%, signifies a considerable backlog, reported to have increased in 73% of UK regions. This has implications on patients with debilitating and often painful MSK conditions.

Since the pandemic the number of elective surgical procedures performed has significantly dropped and is likely to remain so in the short- to medium term. This has implications on the number of CSI referrals, as patients with worsening arthritis, who are eligible for joint replacements but are unable to receive them, are often referred for interim CSI instead offering symptomatic relief while awaiting surgery.^1^ This is also true for patients presenting with non-emergency elective spine pathology, such as disc herniations and scoliosis. These patients are often referred for image guided joint CSI, selective nerve root block, and epidural injections. Issue regarding access to surgical theatres and staffing issues secondary to COVID-19 redeployment^26,27^ has further limited patient access to these treatments. Tackling this challenge needs to be carefully planned, given the existing shortages in the number of MSK radiologists (according to the RCR most recent workforce and staff planning report),^28^ existing staff fatigue and increased sickness leave, as well as shortages of other key staff members including nursing staff, heath-care assistants (HCA) and appointments teams. In addition, availability of rooms and equipment requires careful infrastructure remodelling and probable new investments.^29^

There is little evidence on how CSI may affect vaccine efficacy. It is known that patients receiving multiple repeated corticosteroid treatment for rheumatological or pulmonary disorders are able to generate an adequate antibody response to vaccines.^30,31^ However, there are very limited data on the effect of a single corticosteroid injection on vaccine efficacy. An observational cohort study from the Mayo Clinic reported that intraarticular CSI were associated with of increased risk of influenza infection in patients who had received the influenza vaccine [RR = 1.52 (CI = 1.2–1.93)], compared to a cohort who had not received a corticosteroid injection.^32^ This represented an absolute increase in annual infection risk of only around 1 in 1000. Of note, mean dose-equivalent of methylprednisolone administered with each CSI was 65.9 mg, approximately x1.5 the typical dose administered in the UK for single site injections. Unfortunately, the paper did not report the timeline of steroid injection, immunisation and infection, which would be important to know when interpreting these results. It is not clear if effects on the adaptive immune response and immunological memory correlate with the timing of hypothalamic–pituitary–adrenal (HPA) axis suppression following CSI. Following a single intraarticular corticosteroid injection, the HPA axis and serum cortisol levels are suppressed for 1–4 weeks.^33,34^ Questions still remain with regards to CSI and vaccine efficacy, based on the known timeline of HPA axis suppression following epidural and intraarticular CSI. The consensus from various societies, including the BSSR, suggests consideration of a 2-week interval between any vaccine dose and a CSI procedure, as part of a shared decision-making process with each patient in the context of their clinical indications for injection, as well as the theoretical risk factors for a reduced adaptive immune response to vaccination.^1,4,10^ Long-term follow-up of patients receiving the vaccine, who are also offered CSI, may determine any further considerations to maximise their benefits and reduce any adverse effects.

Our study has some limitations, inherent to the study design. We were unable to capture activity patterns from every NHS trust in the UK. However, widespread geographical representation was achieved from organisations in England, Scotland, and Wales. All questions were binary or designed on a Likert scale, limiting heterogeneity of responses, except for the last free text section, allowing radiologists to highlight any further areas of concern which we may have overlooked. The survey was sent out in November and December 2020, with questions aimed at documenting both the initial lockdown as well as changes in practice throughout 2020. Whilst the questions regarding the initial phase were completed retrospectively, quantitative evaluation could still be achieved by referring to departmental activity. Results can therefore be considered representative of actual events.

## Conclusion

The Covid-19 pandemic represents the biggest challenge facing modern healthcare systems globally. In the UK, national societies and NHS local trusts have provided clear, realistic, and justified evidence-based guidelines to medical professionals delivering CSI services. The BSSR guidelines issued in March 2020, supported by other multidisciplinary guidelines, offered support to musculoskeletal radiologists during these unprecedented times of uncertainty. Current studies support safe resumption of CSI during the pandemic, although cautionmay be prudent regarding vaccination schedules. As the paradigm shifts, a clear strategy is needed to enable radiologists performing CSI to plan and optimise their services towards improved value-based patient care. Government support is needed to avoid NHS trusts being fined for not reaching pre-COVID-19 targets, to enable better use of resources to expand and develop CSI services, amongst others, and enable the NHS to achieve their post-COVID-19 recovery targets.^27^
